# Yellow scorpion (Buthus sinidicus) venom peptides induce mitochondrial-mediated apoptosis in cervical, prostate and brain tumor cell lines

**DOI:** 10.1371/journal.pone.0296636

**Published:** 2024-02-23

**Authors:** Humaira Hassan, Munazza Raza Mirza, Almas Jabeen, Mehtab Alam, Junaid Ahmed Kori, Rabia Sultan, Saeed ur Rahman, M. Iqbal Choudhary

**Affiliations:** 1 Dr. Panjwani Center for Molecular Medicine and Drug Research, International Center for Chemical and Biological Sciences, University of Karachi, Karachi, Pakistan; 2 Dr. Zafar H. Zaidi, Center for Proteomics, University of Karachi, Karachi, Pakistan; 3 Oral Biology, Institute of Basic Medical Sciences, Khyber Medical University, Peshawar, Pakistan; The University of Lahore, PAKISTAN

## Abstract

Scorpion venoms are known to contain over 100,000 biologically active constituents. However, only a few of them have been studied. The major constituents of venom are proteins and peptides, which exhibit various biological and pharmacological properties, including anticancer activities. In the current study, the venom of yellow scorpions (*Buthus sindicus*) found in Sindh, Pakistan, was extracted and evaluated for its anti-cancer and anti-inflammatory activities. The crude venom showed a dose dependent inhibition of phagocyte oxidative burst from human whole blood cells (28.3% inhibition at highest tested concentration of 300 μg/mL). *In-vitro* cytotoxicity of crude venom was evaluated against human prostrate (PC3), cervical (HeLa) and neuroblastoma (U87-MG) cell lines, along with cytotoxicity against normal human fibroblast (BJ) cells. Crude venom was cytotoxic to all cell lines, with prominent inhibitory effect on PC3 cells. Crude venom was fractionated through RP-UPLC, resulted in fifteen fractions, followed by evaluation of their anticancer potential. Among all, the fraction I significantly (P < 0.001) reduced the cell viability of all three cancer cell lines, and exhibited insignificant cytotoxicity against normal cell line. Furthermore, the apoptotic cell death pathway was evaluated for crude venom, and fraction I, in most sensitive cell line PC3, by using flow-cytometry analysis. Both crude venom and its fraction I caused a mitochondrial-mediated apoptosis in prostate cancer cells (PC3). To the best of our knowledge, this is the first report of the anticancer and anti-inflammatory activity of venom of Pakistani yellow scorpions. Results indicate their therapeutic potential, and a case for further purification and validation studies.

## Introduction

Scorpions (class Arachnids, phylum arthropods) are the oldest existing species on earth, for around 430 million years and represent the most venomous class of Arachnidas. Geographically, scorpions are distributed all over the earth, except Antarctica. Scorpions are well-adapted to live in extreme conditions of heat, drought, and starvation for months [1–3]. Over, 1500 species of scorpion have been identified so far. Out of them, the Buthidae family is well known for its hazardous envenomation, which also possesses medicinal importance [4, 5]. The venom is the key to their survival against predators, and competitors. The sting and envenomation generates pain, discomfort, inflammation, and lethal effects, such as muscle paralysis, cardiac arrhythmia, respiratory dysfunction, neurotoxicity, and occasionally death of the victim [6, 7]. One of the mechanisms of scorpion venom is the presence of potent neurotoxins, which inhibits the Na^+^/K^+^-ATPase pump, thus causing the paralysis of parasympathetic and sympathetic nervous systems, with severe-to-fatal consequences [5, 8]. The increased interactions of humans with arthropods due to population growth has increased the incidence of scorpion stinging [9, 10]. Majority of scorpion sting cases are reported from Iran, Saudi Arabia, Turkey, France, China, Italy, and Mongolia due to their climate and conditions. Plenty of literature is available on the taxonomy and epidemiological distributions of scorpions. However, so far literature related to scorpion’s sting cases, and Pakistani scorpion species is limited [11]. Moreover, along with hazards and negative effects, scorpion venom also has therapeutic potential and is currently being investigated by researchers and in the academia and b pharmaceutical industry for drug development [12, 13]. The biological potential of venom depends upon various factors, such as its composition, which varies from species to species, and their geographical distribution [14].

Scorpion venom contains a complex mixture of biologically important molecules, such as mucopolysaccharides, serotonin, phospholipase, hyaluronidase, enzyme inhibitors, histamine, neurotoxins, enzymes (e.g., phospholipase A, phosphodiesterase, acetylcholinesterase, and hyaluronidase), muco- proteins, simple and low molecular weight proteins, along with water and salt [15]. It is estimated that 100,000 different components could be present in the scorpion venom [8]. Toxins of venom are extensively studied because of their pharmacological effect on ion channels as neurotoxins. In addition, disulfide and non-disulfide bridged peptides (NDBP) were also reported in scorpion venom. NDBP are medicinally important components for hemolytic, antifungal, antiviral, electrophysiological, immunological and angiotensin-converting enzyme (ACE) inhibition activities [16, 17]. The mass spectrometric fingerprinting analysis showed low molecular weight peptides are 1/3 of all peptides in the scorpion venom [11]. Such toxins are known for their deleterious effects on organisms. They also display antimicrobial, antimalarial, anticancer, and immunosuppressive activities [18, 19]. As a result, several antifungal, antibacterial, and antiviral substances have been isolated from scorpion venom [20]. In brief, toxins of scorpion venom have contributed to the drug development process [13].

Cancer remains the most fierce disease with the highest mortality rate globally. The current treatments include radiotherapy, chemotherapy, and tumor removal *via* surgery. They also exhibit major side effects and a low success rate. Therefore, the development of safe and effective drugs for cancer prevention and treatment are urgently needed [21]. Proteins and peptides extracted from scorpion venom are commonly employed in cancer treatment [22]. Some of these peptides and proteins bind specifically to cancer cell membranes thus affecting their migration and proliferation [23]. Peptides and proteins, isolated from animal venoms, have also been studied based on their mechanisms. For example, ion channel toxin isolated from scorpion venom that binds specific targets present on cancer cells, or as an angiogenesis inhibitor that are responsible for the permeabilization of cancer cell membranes [24–26].

In several *in-vivo* and *in-vitro* studies on scorpion venom and its toxins, focus on inhibiting the proliferation of cancer through inducing apoptosis and impairing multiple hallmarks of cancers [22]. The efficacy of scorpion venom and toxins have also been tested in various cancer cell lines including neuroblastoma, glioma, lymphoma, breast, pancreatic, prostate. Such studies explored the potential use of scorpion venom and its toxins as anticancer therapeutics [27].

In the current study, we explored the biological potential of crude venom from yellow scorpions (*Bothus sindicus)* (Buthidae family) and its fraction. To the best of our knowledge, this is the first study on the anticancer and anti-inflammatory activities of yellow scorpion found in Sindh, Pakistan.

## Material and methods

### Chemicals

RPMI media, DMEM, fetal bovine serum, PBS buffer were purcahsed from Gibco, USA. Trypsin-EDTA, penicillin–streptomycin solution, antibiotic–antimitotic, trypan blue, DMSO (Dimethylsulfoxide), U Bottom 96-wells plates, and U Bottom 06-wells plates were purchased from Sigma-Aldrich, USA. Phenol red was purchased from Invitrogen, USA. RNA isolation kit, cDNA synthesis kit, and qPCR/Sybr Green master mix, sodium nitrite, 3-(4,5-dimethylthiazol-2-yl)-2,5-diphenyltetrazolium tetrazolium bromide (MTT), 2,7-dichlorodihydrofluorescein diacetate (DCFDA-H2) were purchased from Thermo Fisher, USA, and HPLC grade solvents acetonitrile (ACN), trifluoroacetic acid (TFA).

### Preparation of scorpion venom

Yellow scorpions *(Buthus sindicus)* were collected from the Sindh region of Pakistan. The scorpions were kept individually in glass jars with a regular diet of live locusts, flour beetle larvae, and housefly larvae. The venom was extracted from the scorpion by mild electrical stimulation to the telsons (20 V, 500 mA), followed by collection of venom through capillary tubes. Venom was instantly dissolved in sterile, double-distilled water, and purified by centrifugation at 15,000 rpm for 15 mins. The supernatant was collected, and filtered by using a 0.45-micron filter and stored at -20°C for further analysis.

### Fractionation of venom by UPLC

The mixture of proteins present in soluble venom was uploaded to reverse phase ultra-high-performance liquid chromatography (RP-UPLC) by using C18 reverse-phase analytical column (250×10 mm) purchased from Shimadzu (Japan). The components of crude venom were purified by using a linear gradient of solvent A (water in 0.12% trifluoroacetic acid (TFA) to 70% solvent B (acetonitrile in 0.05% TFA) for 40 mins with a flow rate of 1 mL/min. The fractions were collected manually by monitoring the absorbance at 214 nm. The Qubit Protein Assay kit (Invitrogen, Thermo Fisher Scientific, USA) was used for the quantification of proteins/peptides present in venom, and readings were measured in Qubit 2 fluorometer (Thermo Fisher Scientific, USA).

### Phagocyte oxidative burst detection by chemiluminescence assay

The studies on human whole blood was performed through IRB approved protocol (ICCBS/IEC-008-BC-2015/Protocol/1.0), with informed consents of healthy human volunteers.

Luminol-enhanced chemiluminescence assay was employed, as described by [28]. Briefly 25 μL of (1:20) diluted whole blood in HBSS^++^ (Hanks Balanced Salt Solution, containing calcium chloride and magnesium chloride) (Sigma, USA) was incubated with 25 μL of two-fold increasing concentrations of crude scorpion venom (37.5, 75, 150, and 300 μg/mL), each in triplicate. Control wells received HBSS^++^ and cells, but no compounds. Test was performed in white half area of 96-well plate (Costar, USA), which was incubated at 37°C for 15 minutes in the thermostat chamber of luminometer (Labsystems, Finland). After incubation, 25 μL of serum opsonized zymosan (SOZ) (Fluka, Switzerland) and 25 *μ*L of intracellular reactive oxygen species detecting probe, luminol (Research Organics, USA) were added into each well, except blank wells (containing only HBSS^++^). The level of the ROS was recorded in luminometer in terms of relative light units (RLU).

### Cell lines and cell culture

The cell lines were obtained from Bio bank facility of PCMD (ICCBS) that were purchased from American Type Culture Collection (ATCC): *i*.*e*., HeLa (adenocarcinoma, cervix), PC3 (adenocarcinoma, prostate), U87-MG (human glial cancer cell line), and BJ (normal human skin fibroblast). HeLa, U87-MG, and BJ cells were cultured in Dulbecco’s modified Eagle’s medium (DMEM), supplemented with 10% heat-inactivated FBS, 2 mM L-glutamine, penicillin (100 U/mL) and streptomycin (100 μg/mL). The PC3 cell line was maintained in RPMI-1640, supplemented with 10% (v/v) FBS, penicillin (100 U/mL), streptomycin (100 μg/mL) and 1% of non-essential amino acid. All cell lines were incubated in a T-75 cell culture-treated flask with a 10 mL complete media at 37°C in a humidified incubator with 5% CO_2_ up-to medium.

### *In-vitro* anticancer activity

The effect of scorpion venom and its fractions on cell viability was evaluated by the MTT assay [29]. HeLa cells (3x10^4^/well) and other remaining cell lines (6x10^4^/well) were plated in 100 μL of medium/well in 96-well culture plates, and incubated overnight for recovery and cell adhesion in a humidified atmosphere of 5% (v/v) CO_2_ at 37°C. After incubation, different concentrations of scorpion venom added in each well were. Cells with culture medium only (without scorpion venom) were used as untreated growth control. Three wells were used for each concentration. After 24 hours treatment, 20 μL of 5 mg/mL of sterile MTT (pH 4.7) was added to each well and incubated for another 3 hours. After incubation the supernatant was carefully removed, and 100 μL DMSO was added to each well and shaken for further 15 min at 37°C. The absorbance was measured with a microplate reader at 560 nm (ELISA MRX Revelation Dynex Technologies). The absorbance of untreated cells was considered as 100% growth, and used for viability calculation. The effect of scorpion venom on the viability for human cell lines panel was expressed as the % viability, by using the formula: % viability = A_560_ of treated cells/A_560_ of control cells x 100. The IC_50_ values (venom concentration that causes a 50% reduction of cells) from cancer cells were also determined.

### Total cellular ROS detection by DCFH-DA assay

The intracellular production of ROS in cancerous cells was assayed by using 2, 7- dichlorodihydrofluorescein diacetate (DCFH-DA), as described by Wang *et al*. [30]. At first, PC3, U87-MG and HeLa cells each with cell count of 7×10^4^ cells/well were plated separately in 96-well black, fluorescent plates, and incubated at 5% CO_2_, 37°C in an incubator for adherence. The next day, a fresh stock solution of DCFH-DA was prepared in sterile DMSO. The exhausted media from wells were discarded and FBS-free fresh media with 5 μM of probe concentration were added in the dark, and the plate was left in the incubator for 30 minutes. After incubation, media was removed, and new fresh media with different concentrations of venom and H_2_O_2_ were added. H_2_O_2_ was used as a positive control, and the plate was incubated for 12 hours. After 12 hours, fluorescence was recorded at excitation 485 nm, and emission at 520 nm.

### Genes expression (*p53*, *bax*, *and bcl-2*) analysis in PC3 cells

PC3 cells (5×10^5^ /well) were seeded on 6-well plate, and incubated for 24 hours. After incubation, the concentration of scorpion venom (300 μg/mL) and fraction I (200 μg/mL) with fresh medium in duplicate wells were added, and incubated for 24 hours. The wells without any treatment were considered as negative controls. After incubation, cells were harvested for RNA isolation, followed by reverse transcriptase PCR (RT-PCR) (Thermo Fisher Scientific, USA). Total RNA was isolated from cells by using Revert-Aid RNA purification kit according to the manufacturer’s specifications (Invitrogen, USA). The concentration of total RNA in final elutes was determined through Nano drop. cDNA was synthesized from extracted RNA (1 μg) by using cDNA synthesis kit (Invitrogen, USA) in a 20 μL volume reaction. The qPCR for targeted *p53*, *bax*, and *bcl-2* genes was performed in triplicates using SYBER green Master Mix kit (Invitrogen, USA). GAPDH (glyceraldehyde 3-phosphate dehydrogenase), a housekeeping gene, was considered as internal control. The primer sequence for RT-PCR were 5´-CTCTCTGCTCCTCCTGTTCG-3´ and 5´-TGGAATTTGCCATGGGTGGA-3´ for *GAPDH*; 5´- ACCCAGGACTTCCATTTGCT-3´ and 5´- AGGTGGTTTCAAGGCCAGAT-3´ for *p53*; 5´- GGACGAACTGGACAGTAACATGG-3´ and 5´- GCAAAGTAGAAAAGGGCGACAAC-3´for *Bax*; 5´- CGCCCCTGGTGGACAA-3´, and 5´- GTCACGGTCTGCCACG-3´ for *Bcl-2*. Fold change expression of each gene was determined by 2^-**∆∆Ct**^ in comparison of the threshold cycle (CT) value of the gene of interest to the CT value of the selected internal control (GAPDH). Amplified PCR products were then subjected to electrophoresis at 70V in 1.5% (w/v) agarose gel for 1.5 hours. A 100 bp DNA ladder was used as a molecular marker. The gels were examined, and the intensity of each band was measured by using Gel Doc imaging system UVIsave D-55/20M version 15.08 (UVItec, England). The expected size of the amplified PCR products was as 254 bp for *GAPDH*, 405bp for *Bcl-2*, 150bp for *Bax*, and 358bp for *p53*.

### Flow cytometry analysis

Flow cytometry analysis was carried out to distinguish live/apoptotic/necrotic cells with Annexin V-FITC/7AAD and PI stainings (Invitrogen, Thermoscientific, USA). In brief, PC3 cell line (4×10^5^ cells) were seeded in 6-well plate, and plate was incubated for 24 hours. The cells were treated with 200 μg/mL of fraction I, for 24 hours. After treatment, cells were harvested, and washed three times with iced-cold phosphate buffered saline (PBS). Cells were stained with an Annexin V-FITC/7AAD and PI in dark for 10 mins, followed by termination of reaction with 500 μL of binding buffer and cells were incubated on ice. Stained samples were analyzed on a flow cytometer (Dako Cytomation Cyan LX, Dako Corp. USA) and 10,000 events were collected. The data were then plotted using Summit V4.3 Build 2445 software (Dako Colorado Inc., USA).

### Statistical analysis

All data was collected from at least three independent experiments and expressed as means ± standard deviation (SD). Statistical analysis was performed by SPSS statistics software, applying one-way ANOVA and Tukey post hoc test. Statistical significance indicated * was considered as significant (p ≤ 0.05), whereas ** = p ≤ 0.01, and *** = p ≤ 0.001.

## Results

### Oxidative burst inhibitory activity of scorpion crude venom

**Fig 1:**
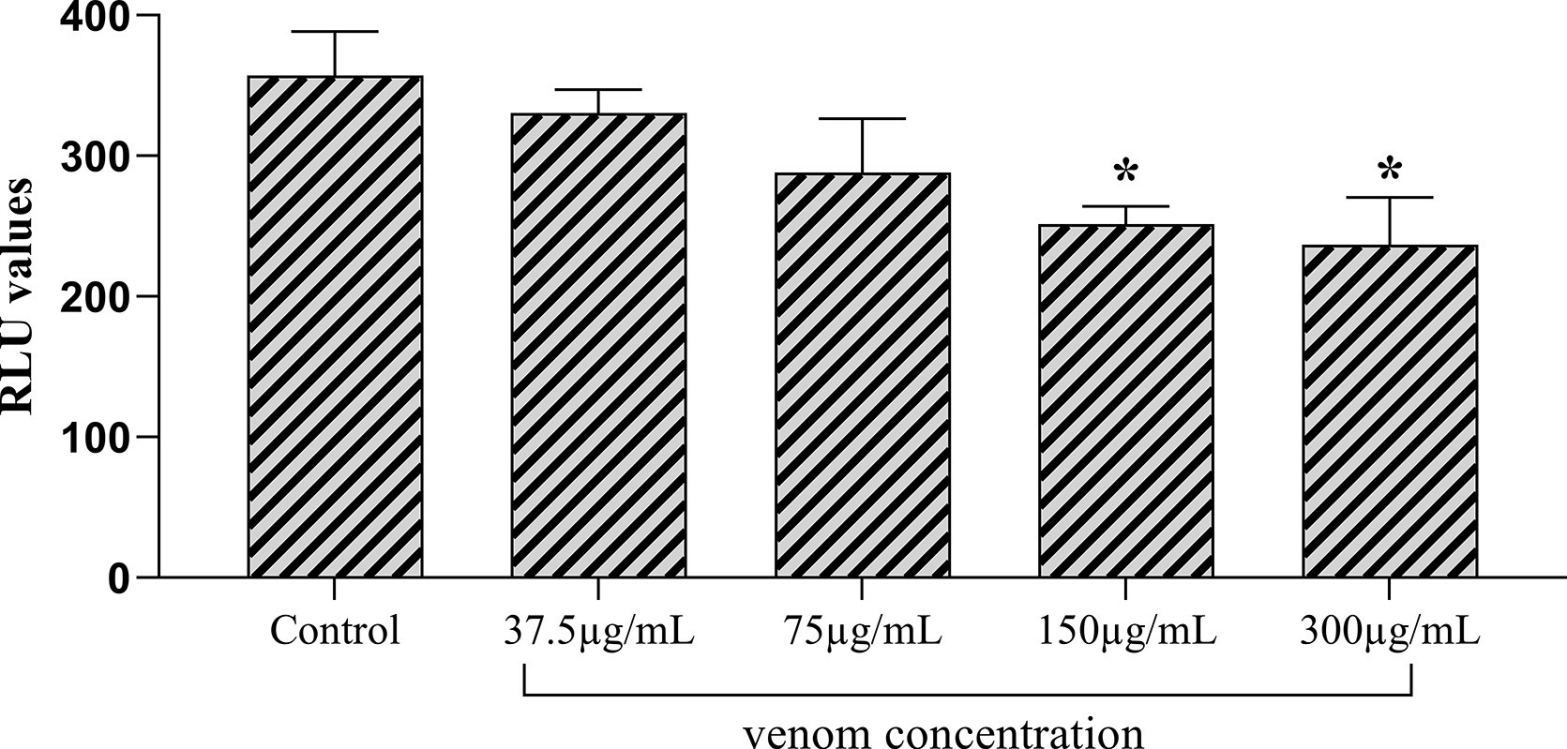
The bar graph represents the effect of different concentrations of crude venom on oxidative burst. Significant decrease in ROS was observed at 150 and 300 μg/mL concentrations. All assays were performed in triplicate, and the data is presented as mean±SD. The *p* values were determined by comparing the control group *versus* every group of treatment by one-way annova, tukey post hoc test. Significant differences represented as *p<0.05, ** p<0.01, ***p<0.001.

The crude scorpion venom was evaluated for inhibition of myeloperoxidase dependent reactive oxygen species (ROS) which were generated by human whole blood phagocytes in response to zymosan treatment. Overexpression of ROS in one of the mechanism of inflammations. The crude venom showed a dose dependent inhibitory effect with moderate level of inhibition (28.3%) at highest tested concentration of 300 μg/mL (Fig 1).

### *In-vitro* cytotoxicity of scorpion crude venom against cancer cell lines

**Fig 2:**
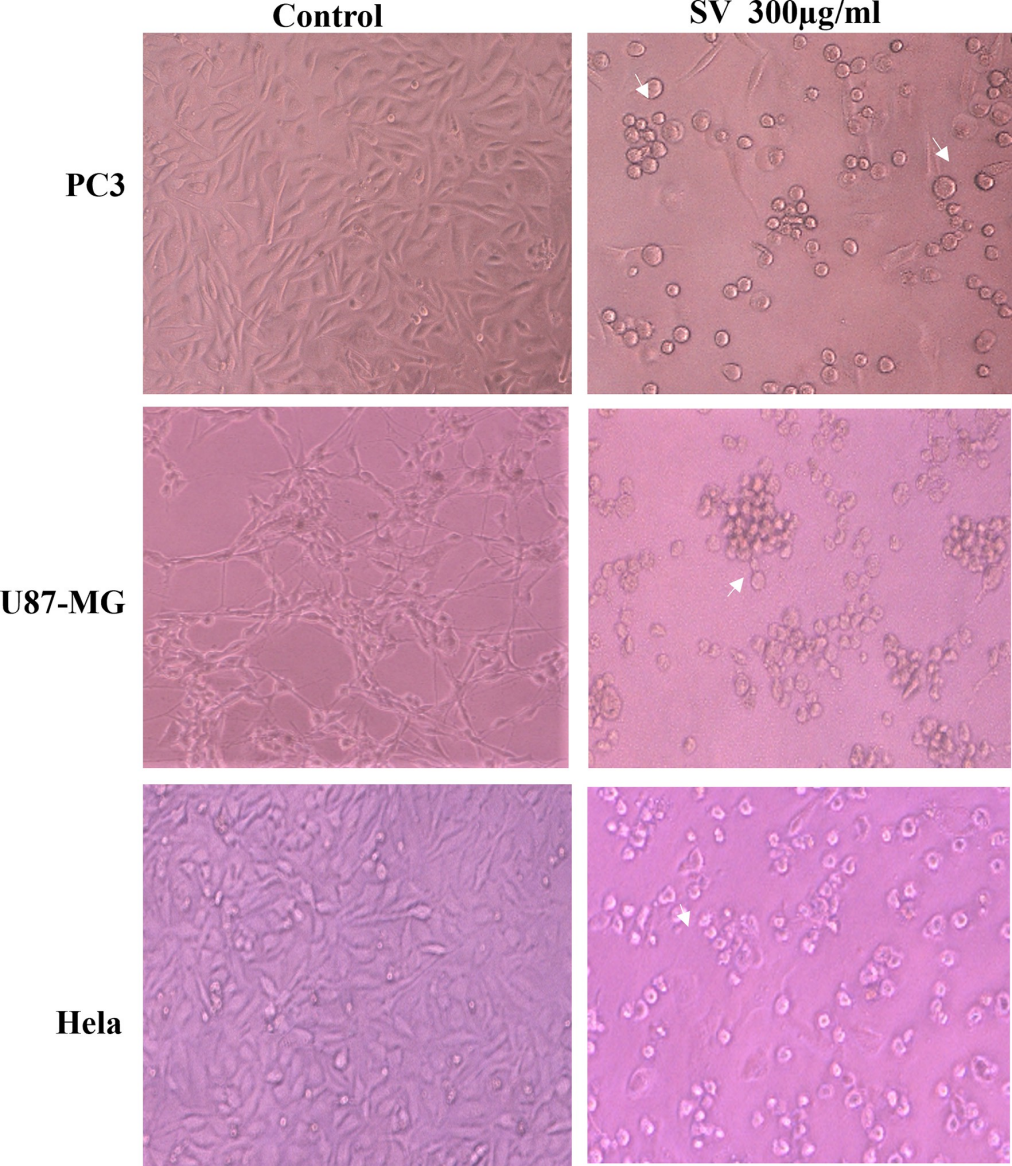
MTT reduction in PC3, HeLa, U87 and BJ cells after exposure for 24 hours with different concentrations of rude scorpion venom. All assays were performed in triplicate, and the data is presented as mean±SD. The *p* values were determined by comparing the control group *versus* every group of treatment by one-way annova, and tukey post hoc test. Significant differences represented as *p<0.05, ** p<0.01, ***p<0.001.

**Fig 3:**
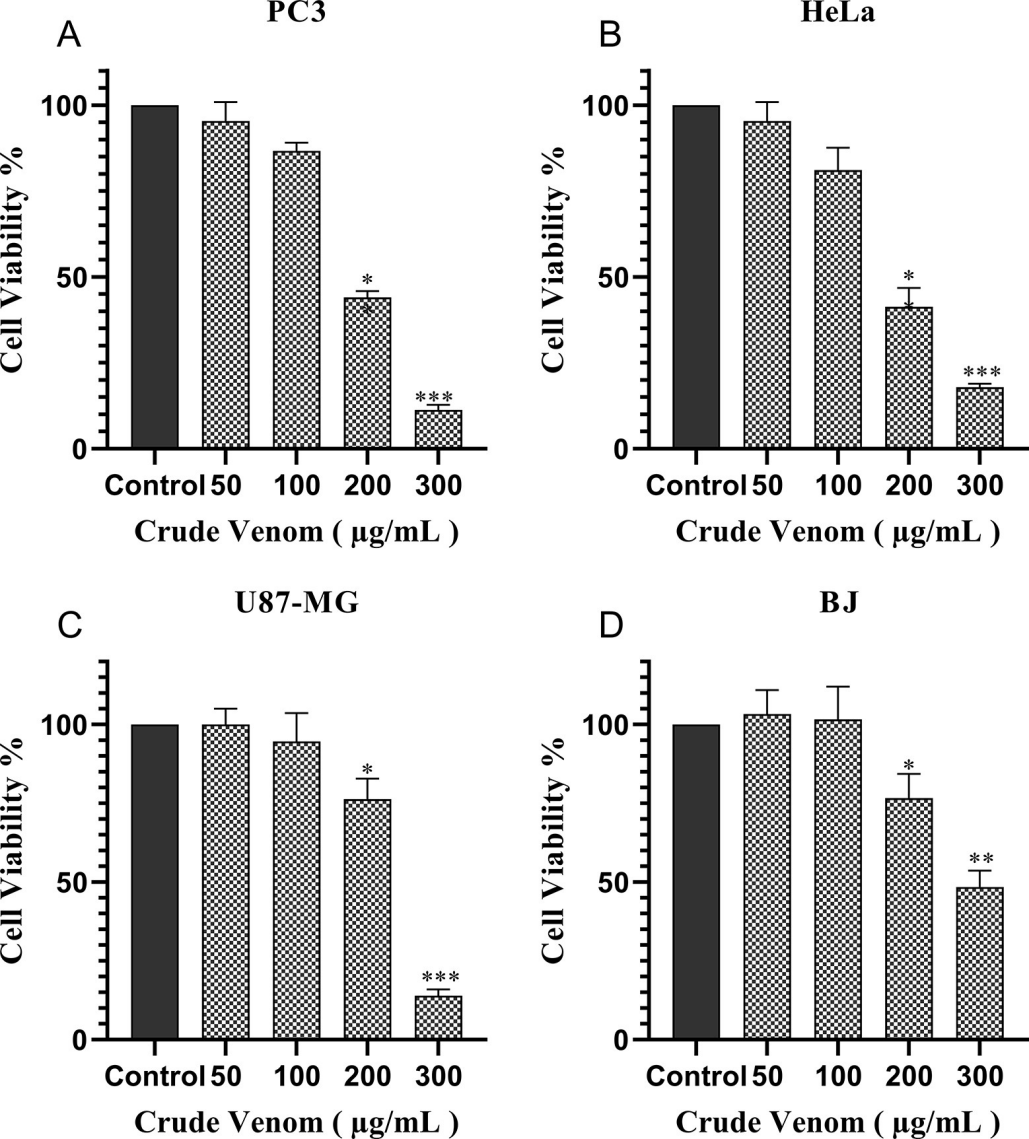
The morphological alterations of U87-MG, PC3, and HeLa cells after treatment with scorpion venom for 24 hours were observed. Control cells without venom showed normal fibroblast morphology and after treatment with venom, cells exhibited abnormalities in shape (white arrow), aggregation, swelling, and rupture membrane. Original magnification 10X (phase contrast).

The cytotoxic effect of crude scorpion venom was tested against a panel of cancer cell lines, including U87-MG, PC3, and HeLa. Fig 2 showed the crude scorpion venom induced morphological alterations and decreased cell viability of all the tested cell lines at 300 μg/mL concentrations. The cell viability of PC3, HeLa, and U87-MG at venom concentrations of 100, 200, and 300 μg/mL, decreased significantly to 87% (*p* <0.05), 42% (*p* <0.01), and 9.9% (*p* <0.001) respectively, suggesting a dose-dependent inhibitory effect of scorpion venom (Fig 3A–3C). Moreover, no significant cytotoxic effect at tested crude venom concentrations of 200 and 100 μg/mL was observed on normal BJ cell line (Fig 3D). The IC_50_ values of cytotoxicity of venom on studied cell lines are presented in Table 1. Notably, the optimum time for cell incubation with venom was 24 hours, as the anti-proliferative effect reduced at a longer time.

**Table 1:** IC_50_ Values of scorpion venom treatments for 24 hours against cancerous cell lines. Values represent the mean ±SD obtained from three independent experiments.

Cell lines	Crude Scorpion Venom IC_50_ (μg/ml)	Percent Inhibition % (300–100 μg/ml conc)	Fraction I IC_50_ (μg/ml)	Doxorubicin* IC_50_ (μM)
HeLa (Cancer)	171.7 ± 1.04	82.1–18.9	---	1.1± 0.04
PC3 (Cancer)	170.8 ± 0.78	86.6–13.3	128.8 ± 1.08	1.4± 0.05
U87-Mg (Cancer)	237.4 ± 0.95	86.1–5.3	---	---
BJ (Normal)	293.2 ± 0.69	51.5- -1.6	---	0.15± 0.03

*Doxorubicin was used as a standard drug.

### Cytotoxicity of fractions

**Fig 4:**
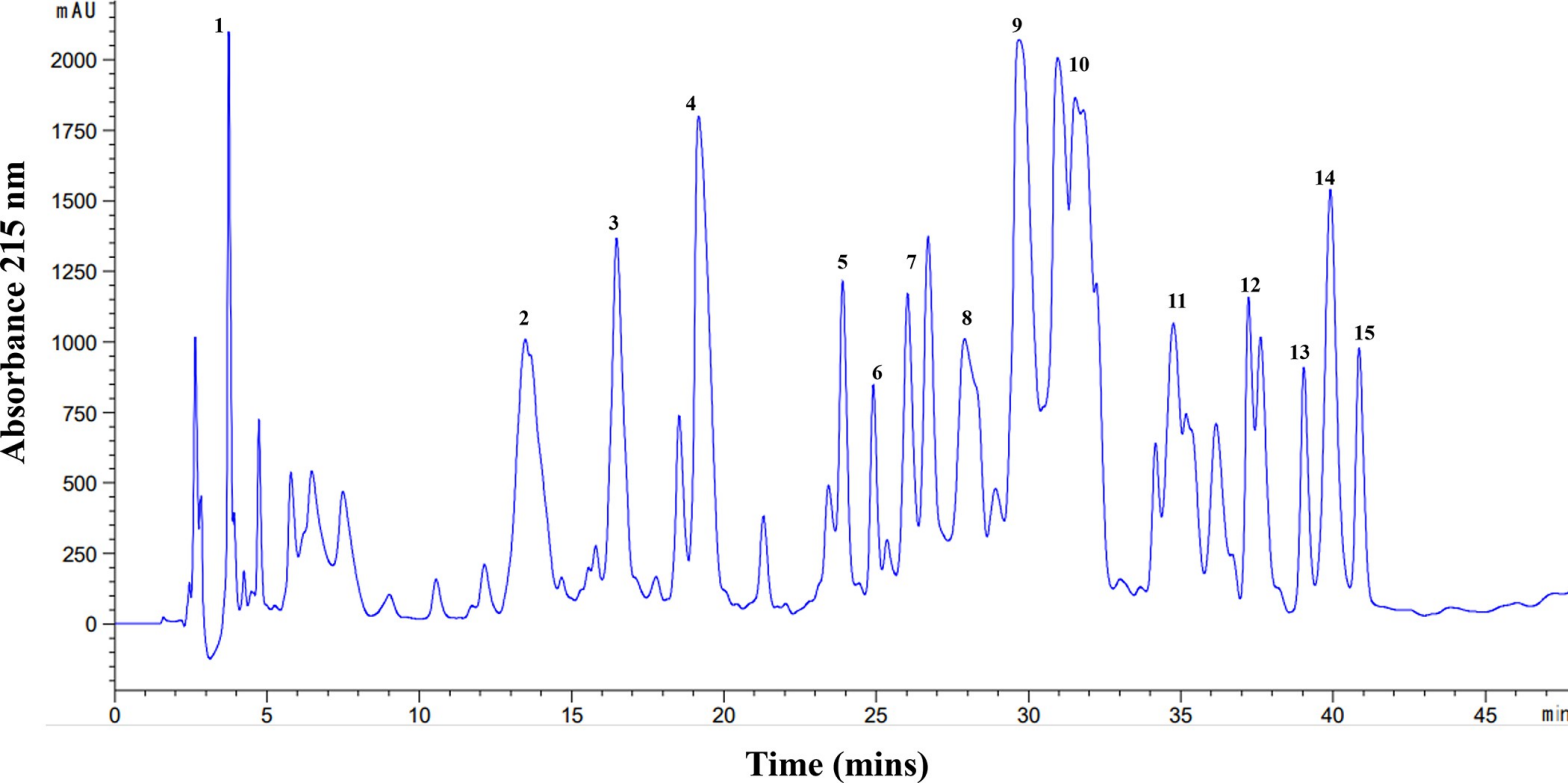
UPLC separation of venom. Soluble venom isolated from Scorpion was applied to the UPLC system using a C18 reverse phase column and eluted with a linear gradient of solvent A (0.12% of trifluoroacetic acid in water) to 70% solvent B (0.10% TFA in acetonitrile) for over 50 min.

**Fig 5:**
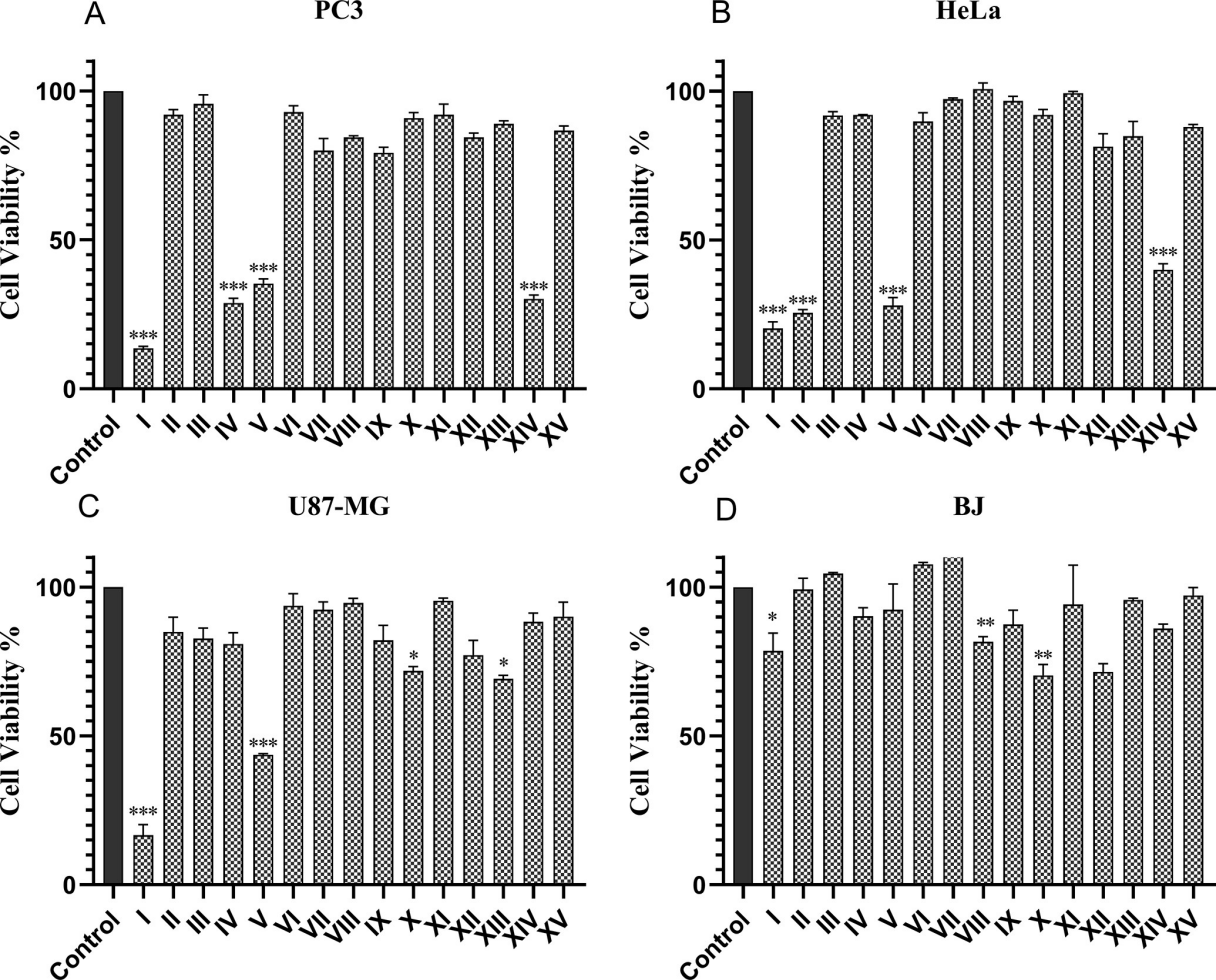
Cytotoxicity evaluation of UPLC fractions of scorpion venom. The fractions were assayed on three different cancerous cell lines PC3, HeLa, U87-MG, and BJ normal cell line. The *p* values were determined by comparing the control group versus every group of treatment by one-way annova, and post hoc tukey test. Significant differences are presented as *p<0.05, ** p<0.01, ***p<0.001.

In order to achieve a certain level of purification, fractionation of scorpion venom by RT-UPLC was initially performed (Fig 4). Venom was eluted in 15 independent fractions at different time intervals, followed by evaluation of their cytotoxicity against cancer cell lines PC3, U87, and HeLa, and normal Human skin fibroblast cell line BJ. Fractions I, V and XIV significantly decreased the cell viability of all cancer cell lines (*P*-value<0.05), whereas fraction II, caused a decrease in viability by 25% (*P*-value<0.05) in the HeLa cell line, whereas fraction IV was found to have activity against PC3 cell line. Fraction IV caused a decrease in cell viability (*P*-value<0.05) of the PC3 cell line. Fractions that have shown cytotoxicity against cancer cell, but lines did not show any significant toxicity to normal BJ-cells (*P*-value>0.05) (Fig 5). Among all the fractions, fraction I was found to be the most active against various cell lines. Moreover, in PC3 cell line, fraction I showed an IC_50_ of 126.2 μg/mL, even less then the IC_50_ value of the crude venom (IC_50_ ± 170.8 μg/mL) (Table 1).

### Mitochondrial ROS measurement in PC3 cell

**Fig 6:**
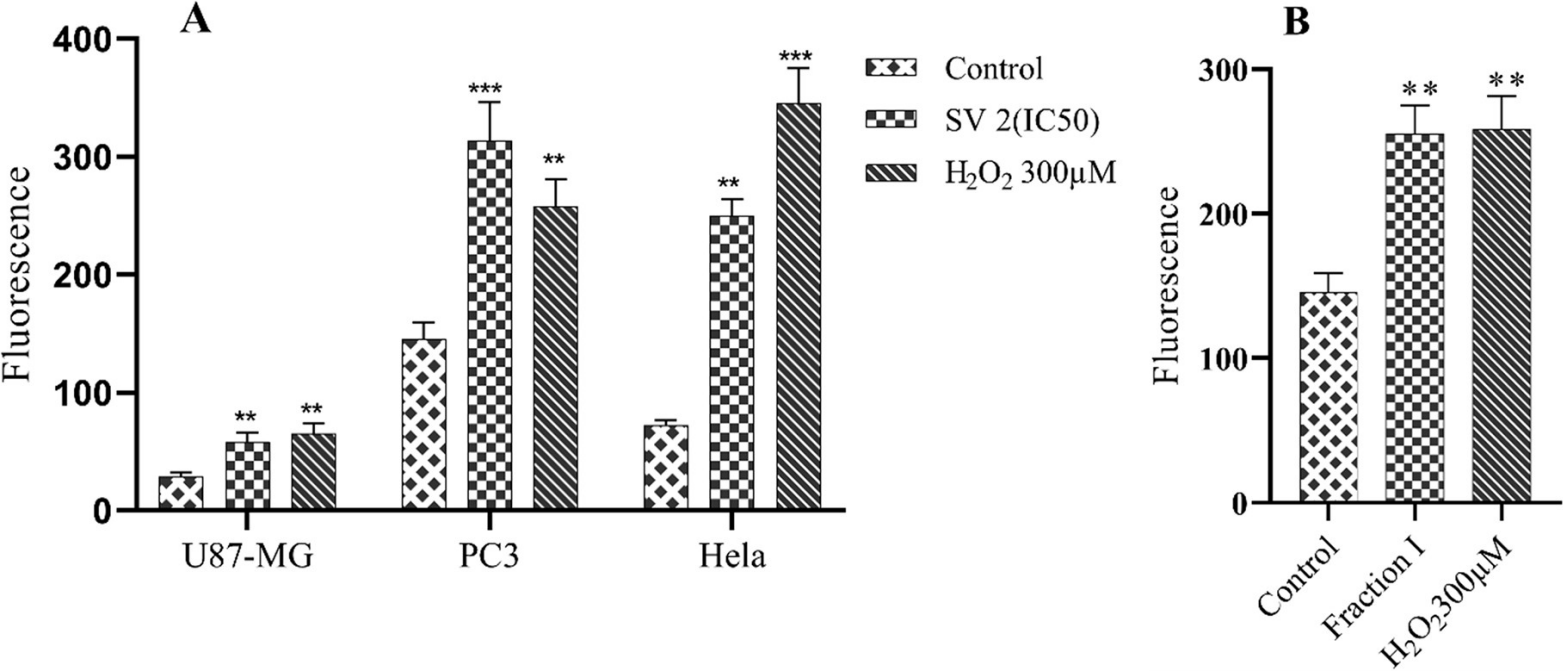
Increased reactive oxygen species (ROS) production after treatment with Scorpion Venom (SV) twice IC_50_ value against U87-MG, HeLa, and PC3 cells (A), and Fraction I with a concentration of 200 μg/mL against PC3 cells (B) after 12 hours of incubation. All assays were performed in triplicate, and data is represented as mean±SD. **P-value<0.01, ***P-value<0.001 different levels of significance as compared with the negative control cells.

Scorpion venom with concentration twice of the IC_50_ value (300 μg/mL) caused the production of ROS in all cell lines (*P*-value<0.05) after 12 hours of incubation (Fig 6A). However, PC3 cell line was found to be the most sensitive (IC_50_ 170.8 μg/mL) in case of crude venom effect as well as for its fractions, therefore, PC3 cell line was selected for downstream mediatory assays. Fraction I significantly induced (*P*-value<0.05) ROS production in PC3 cell line at a concentration of 200 μg/mL, as compared to negative control (Fig 6B).

### Effect of scorpion venom and fraction I on apoptosis-related genes in PC3 cells

**Fig 7:**
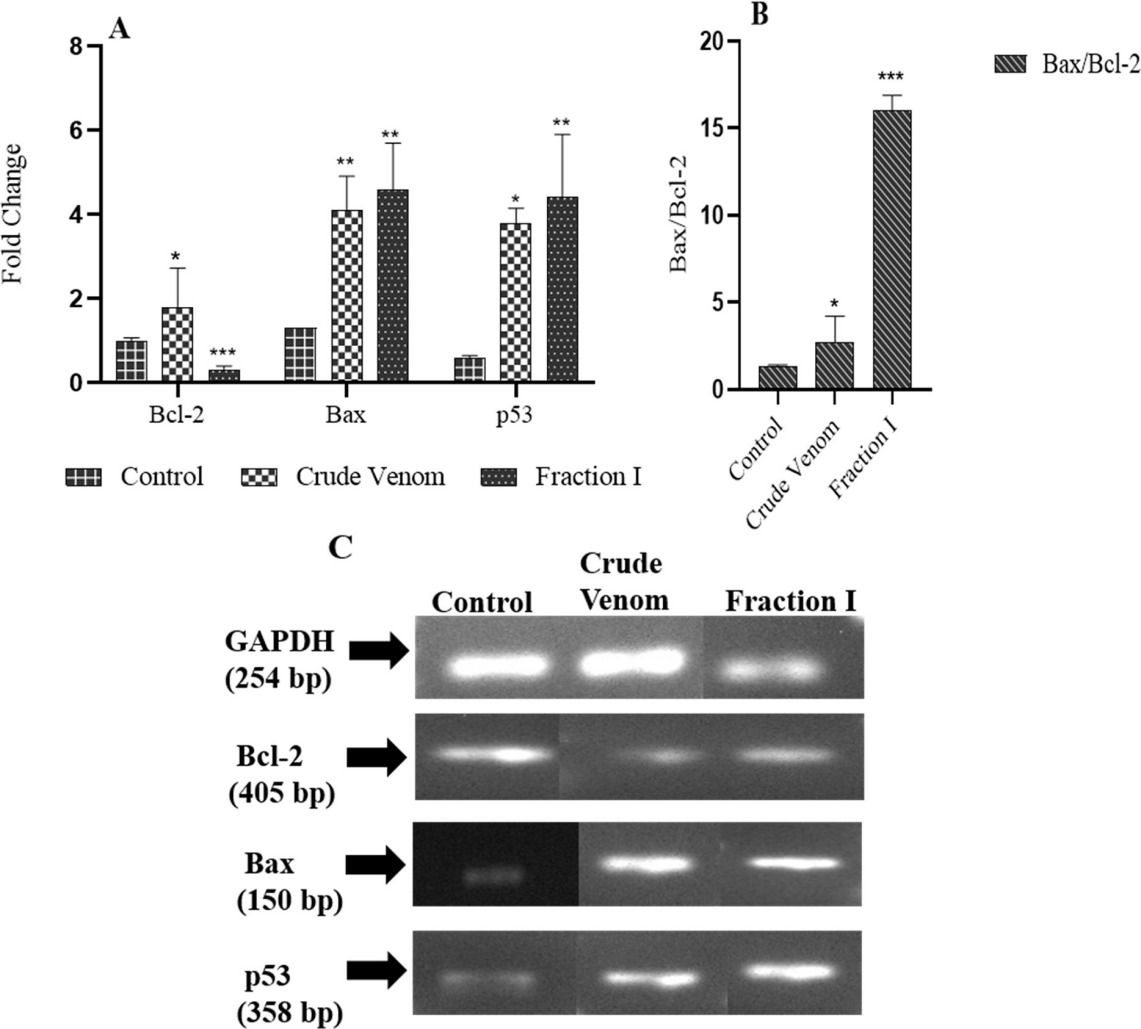
Effect of fraction I and crude venom treatment on *p53*, *Bax* and *Bcl-2* genes expression in PC3 cells. The mRNAs expressions of *p53*, *Bax* and *Bcl-2* were detected by RT-PCR after 24 hours of incubation. Relative intensities of targeted genes ratio mRNAs expression levels were compared with GAPDH (A). (B) represents the Bax/Bcl-2 ratio. Values represent the mean+SD obtained from at least three independent experiments. The *p* values were obtained comparing the control group *versus* treatment groups by one-way annova, and post hoc tukey test. Significant differences are represented as *p<0.05, ** p<0.01, ***p<0.001.

The effect of scorpion venom (300 μg/mL) and fraction I (200 *μ*g/mL) on PC3 cells after 24 hours treatment was evaluated through the expression levels of *bcl-2*, *Bax*, and *p53* apoptosis-related genes, while *GAPDH* was the internal control (Fig 7A). A significant fold (p<0.05) decrease in expression of *Bcl-2* was caused by Fraction I, as compared to untreated control. Whereas, no significant effect of crude venom at the expression of *Bcl-2* was observed. A significant increase (p<0.01) in fold change expression of *Bax* and *p53* genes were also observed after treating cells with both crude venom and fraction I, as compared to untreated control groups. For crude venom, a significant increase (p<0.05) in *Bax/Bcl-2* ratio was entirely due to the overexpression of Bax. Whereas, fraction I more significantly (p<0.001) increased the *Bax/Bcl-2* ratio, as compared to control and crude venom, due to the increased expression of *Bax* and decreased expression of *Bcl-2* (Fig 7B).

### Flow cytometry analysis

**Fig 8:**
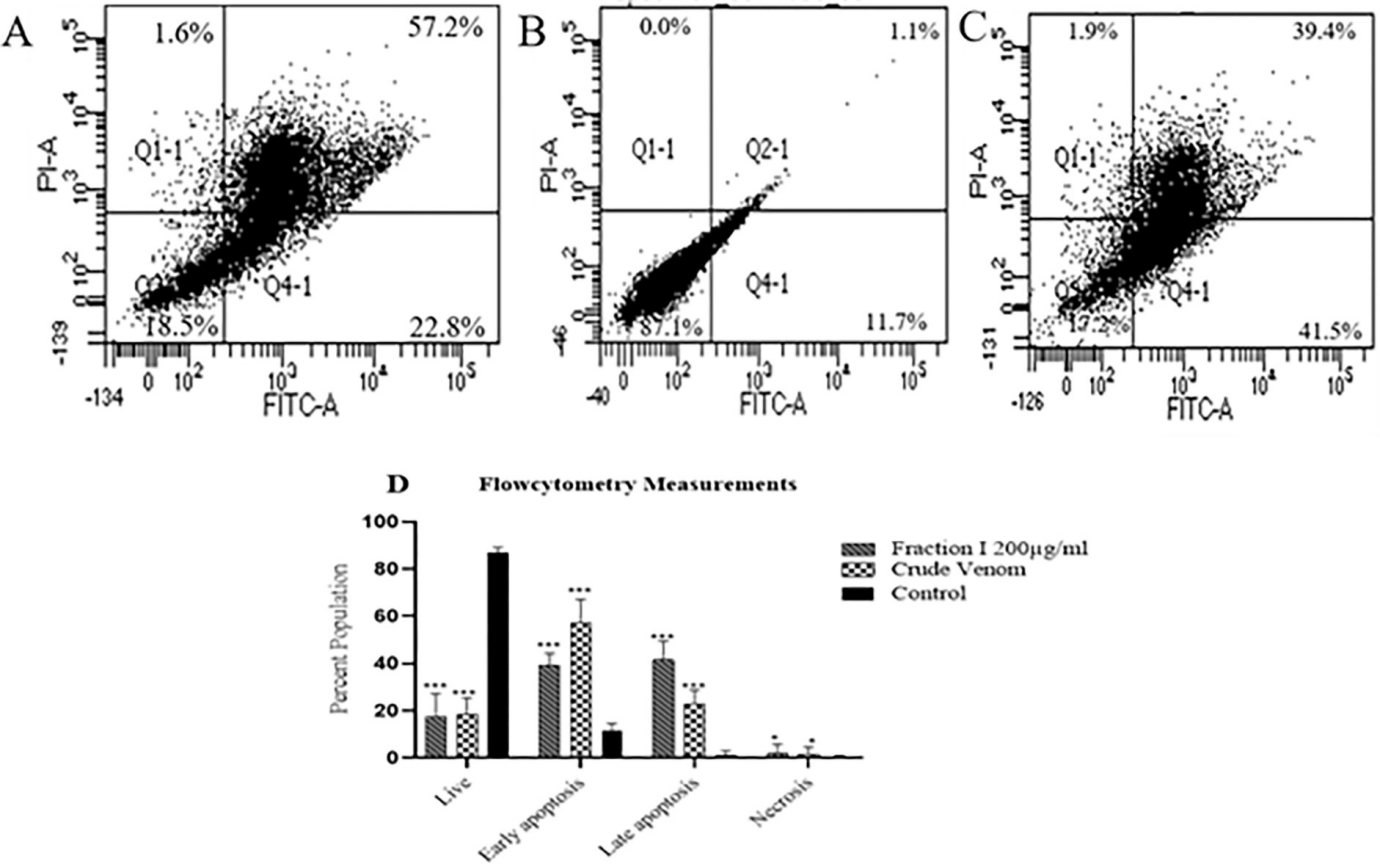
Treated PC3 cells with twice IC_50_ value of crude scorpion venom (A) Apoptosis and necrosis ratio of PC3 cells in untreated negative control cells (B), treated PC3 cells with 200 μg/mL of Fraction I (C). The figure (D) Shows the graphical representation of flow-cytometry measurements from 3 independent experiments and represented as the mean±SD. **P-value<0.01, ***P-value<0.001 represents different levels of significance compared with negative control cells (D).

To investigate the apoptotic cell death flow-cytometry technique were used. The PC3 cells were treated with twice the IC_50_ of crude scorpion venom, and 200 μg/mL of Fraction I for 24 hours, while untreated live cells were used as negative control. As shown in Fig 8, the percentage of early and late apoptosis was significantly increased (p<0.001) in crude venom, and Fraction I treated groups, when compared to the untreated control groups. Whereas, the ratio of early apoptosis was more in case of crude venom, as compared to fraction I, whereas fraction I has a higher ratio of late apoptosis, as compared to crude venom, suggesting scorpion venom and Fraction I induced apoptosis in cancer cell.

## Discussion

Natural compounds have been used for the treatment of many diseases since ancient times. During search for new bioactive agents of natural origin, animal toxins have also proven to be valuable source of new drug candidates [23]. The present work was aimed to explore the biological potential venom components of yellow scorpion, found in the rural areas of Sindh, Pakistan. To evaluate the biological potential of crude scorpion venom, the cytotoxicity was evaluated against three different cancer cell lines namely U87-MG, PC3, HeLa, and normal cell line BJ by MTT assay The scorpion venom significantly reduced the proliferation of cells in all three cancer cell lines. After 24 hours of venom exposure cell lines have shown a ranging degree of viability. This may be due to the active compounds present in the venom. Cytotoxicity variations that have occurred after 24 hr exposure of venom to cells might be due to the complex composition of venom, and targets that are being expressed on cancer cells [26]. Our observations were found to be consistent with earlier studies on other species of scorpions, such as *Androctonus australis*, *Rhopalurus junceus*, and *Odontobuthus doriae* which also showed a selective and differential cytotoxicity against many human malignant cell lines [19, 31, 32]. In addition, scorpion venom reduced the cell viability of the glioblastoma cell line (U87-MG), and showed cytotoxicity against normal cell line BJ at a higher concentration. These results are in agreement with previous studies where BMK venom has shown a significant cytotoxicity towards tumor brain cell line U251-MG while it was not toxic to the normal cells (BJ) [33].

To reduce the complexity of venom, crude venom was fractionated by UPLC, and 15 independent fractions were collected. The cytotoxicity of each fraction with concentration of 200 μg/mL was evaluated against all three cancer cell lines, and normal BJ, as mentioned above. Fractions I and V have significantly reduced the cell viability of all cancer cell lines. However, fraction I was found to be the most potent with minimum IC_50_ value in PC3 cell line. Fraction XIV was found active against PC3 and HeLa cell lines, Fraction II against HeLa, and Fraction V was found to be active against PC3 only. Interestingly, the fractions that are active on cancer cells did not show any cytotoxicity to normal BJ cell line. The findings of our study were similar to the study by Béchohra *et al*, in which they collected two toxic fractions FtoxG-50 and F3 from *Androctonus australis* scorpion, and F3 showed no cytotoxic effect against a normal cell line [34]. The difference in the cytotoxic effect of each fraction against cancer cell lines and normal fibroblastic cells suggested the presence of different peptides in fractions, and cellular targets they interacted.

Additionally, the cytotoxicity were confirmed by the ROS measurement. As ROS are considered to be the key regulators of cellular oxidative stress, and play a significant role in tumor cell damage and mitochondrial stability. An imbalance of redox levels may cause damage major components of cells, such as DNA, protein, lipids, and membranes which ultimately leads to oxidative stress and cell death [27]. Recent studies have also shown that the role of ROS in cancer is not limited to genotoxicity and mutagenic effects that may initiate cancer. ROS may also enhance either proliferation or death of cancer cells, depending on the intracellular and exogenous environment. Therefore, the role of ROS in inducing cancer cell death may form the basis of ROS-generated antineoplastic therapies, [27]. Thus to determine whether the apoptosis against cancer cell lines was induced by ROS production, the intracellular quantification of ROS by DCF fluorescence was measured. The results obtained showed a significant increase in ROS production induced by crude venom at a concentration of twice the IC_50_ for 12 hours against all cell lines. Fraction I was tested against the PC3 cell line, and showed a significant increase in ROS production at 200 μg/mL concentration after 12 hours of incubation. These results suggest that ROS may be a key early signal used by scorpion venom and fraction I to activate the apoptosis. Similar to the results of the current study, toxins from various animals have also been reported to induce intrinsic apoptosis through the up-regulation of ROS [34].

Next we assessed the expression of genes involved in the regulation of apoptosis. As the p53 mechanism of cell death acts as a transcriptional regulator, and may influence the activation of pro-apoptotic genes like *Bax*, and suppression of anti-apoptotic genes *Bcl-2*. To resist apoptotic cell death, cancer cells have developed various mechanisms [35]. The over-expression of the anti-apoptotic *Bcl-2* family of proteins and suppression of the *Bax* gene are among them [26]. Crude scorpion venom and its fraction I induce mitochondria-mediated apoptosis by upregulating p53 expression, leading to an increased *Bax/Bcl-2* ratio, which activates caspases and dominates intrinsic apoptotic pathways.

The process of apoptosis is also associated with DNA fragmentation, loss of the mitochondrial membrane integrity, cell surface exposure of phosphatidylserine, and apoptotic body formation. It should be noted that the externalization of phosphatidylserine on the cell membrane provides an early apoptotic occurrence, and can be detected by annexin-V staining. In this respect, counterstaining with PI can provide a distinction between apoptosis and necrosis [36]. The results obtained showed that scorpion venom and its fraction I had induced a higher rate of apoptosis, as compared to the negative untreated control. In conclusion, venom of the Pakistani yellow scorpion has the potential to slow down the progression of cancer cells by inducing apoptosis *via* ROS production, as well as the release of pro-apoptotic signals. As a comparative study, we run experiments in parallel with crude venom, and its most potent fraction I against sensitive cancer cell line PC3. The results showed that fraction I caused mitochondrial mediated apoptosis leading to cancer cell death with more efficiency. To our knowledge, this is the first study demonstrating the anticancer potential of Pakistani scorpion venom, and its fractions with cell death events.

## Conclusion

In summary, the present work demonstrated the biological activity of crude venom from yellow scorpions (*Buthus sindicus*). and its UPLC fractions Both crude venom and its fractions showed anti-cancer potential on epithelial and glioma cancer cell lines with minimum cytotoxicity against normal cell lines. The anticancer activity of the fraction I was found to be the most potent among all fractions, and as compared to the crude venom with minimum IC_50_ reduce against PC3 cell line. This indicate that relatively purified form of the venom may produce more specific anticancer activity. Furthermore, it was observed that the crude venom and fraction I can trigger the mitochondrial-mediated apoptotic cellular death, along with the production of reactive oxygen species (ROS) in cancer cells. The crude venom also showed a moderate anti-inflammatory potential by inhibiting phagocyte oxidative burst from human whole blood cells. To the best of our knowledge this is the first report of the therapeutic potential of crude venom and its fractions, isolated from yellow scorpion (*Buthus sindicus*), found particularly in Sindh, Pakistan. The study forms the basis for further investigation on the venom fractions to develop pure protein / peptide based anticancer drug candidates.

## Supporting information

S1 Raw image The original / uncropped agarose gel electrophoresis of *GADPH*, *p53*, *Bax* and *Bcl-2* showing the product sizes compared to 100bp DNA marker. The intensity of each band was measured by using Gel Doc imaging system UVIsave D-55/20M version 15.08 (UVItec, England). The expected sizes of the amplified PCR products were 254 bp for *GAPDH*, 405bp for *Bcl-2*, 150bp for *Bax*, and 358bp for *p53*. The lanes labelling is mentioned in the image.
(PDF)
