# Supplementary material for: Yellow scorpion (Buthus sinidicus) venom peptides induce mitochondrial-mediated apoptosis in cervical, prostate and brain tumor cell lines
Source: PLoS One. 2024 Feb 23;19(2):e0296636. doi: 10.1371/journal.pone.0296636 (PMC10890731; doi:10.1371/journal.pone.0296636)

Supplementry Information File

Yellow Scorpion (Buthus sindicus) Venom Peptides Induce Mitochondrial-Mediated Apoptosis in Cervical, Prostate and Brain Tumor Cell Lines

Humaira Hassan[1], Munazza Raza Mirza[1*], Almas Jabeen[1*], Mehtab Alam[2], Junaid Ahmed Kori[1], Rabia Sultan[1], Saeed ur Rahman[3], M. Iqbal Choudhary[1]

1 Dr. Panjwani Center for Molecular Medicine and Drug Research, International Center for Chemical and Biological Sciences, University of Karachi, Karachi-75270, Pakistan.

2 Dr. Zafar H. Zaidi, Center for Proteomics, University of Karachi, Karachi-75270, Pakistan.

3 Oral Biology, Institute of Basic Medical Sciences, Khyber Medical University, Peshawar, Pakistan.

Fig S1: The original / uncropped agarose gel electrophoresis of *GADPH*, *p53, Bax* and *Bcl-2* showing the product sizes compared to 100bp DNA marker. The intensity of each band was measured by using Gel Doc imaging system UVIsave D-55/20M version 15.08 (UVItec, England). The expected sizes of the amplified PCR products were 254 bp for *GAPDH*, 405bp for *Bcl-2*, 150bp for *Bax,* and 358bp for *p53*. The lanes labelling is mentioned in the image.

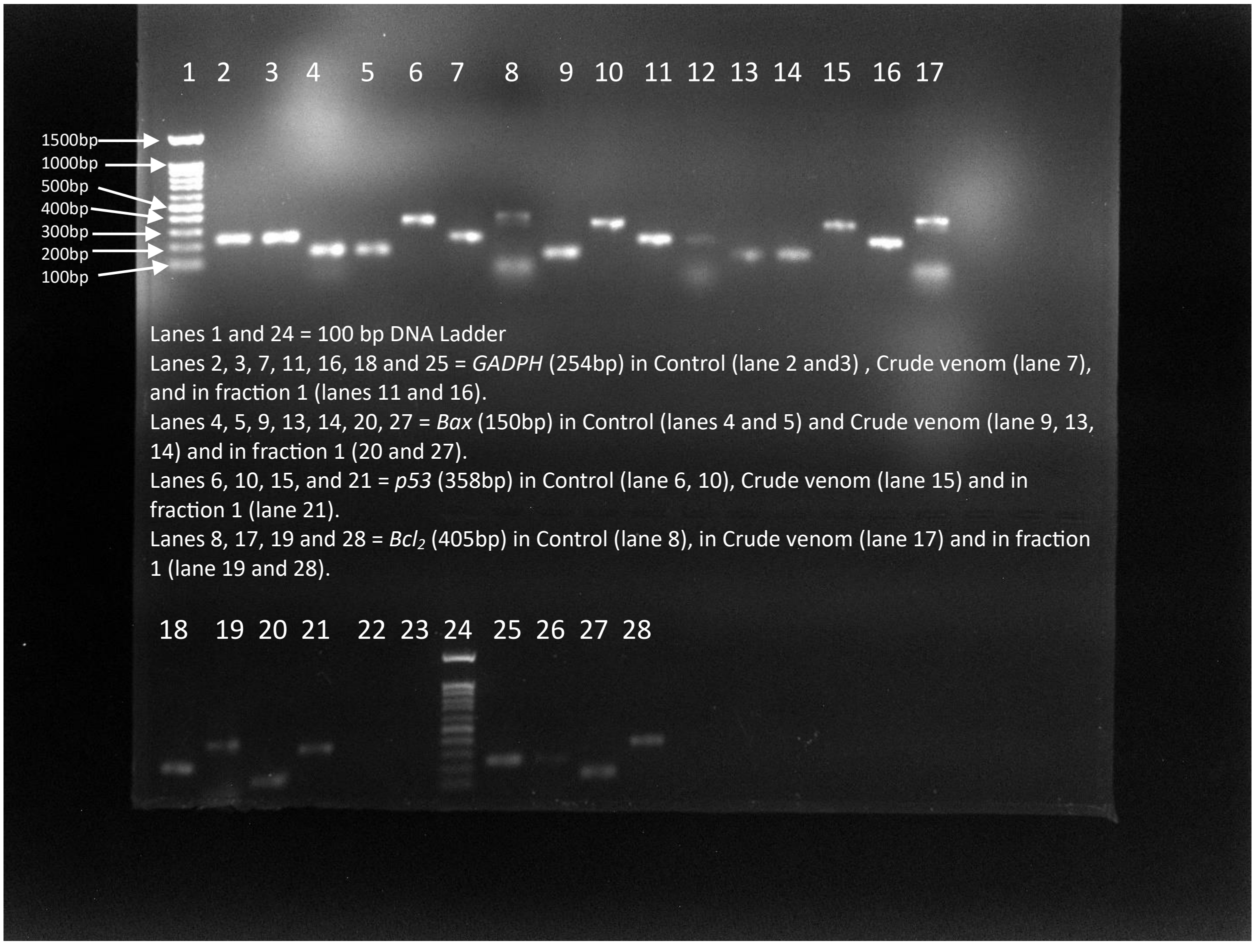

Supplement: S1 Raw image — The intensity of each band was measured by using Gel Doc imaging system UVIsave D-55/20M version 15.08 (UVItec, England). The expected sizes of the amplified PCR products were 254 bp for GAPDH, 405bp for Bcl-2, 150bp for Bax, and 358bp for p53. The lanes labelling is mentioned in the image. (PDF) [file pone.0296636.s001.pdf]
